# Clubroot resistance derived from the European *Brassica napus* cv. ‘Tosca’ is not effective against virulent *Plasmodiophora brassicae* isolates from Alberta, Canada

**DOI:** 10.1038/s41598-021-93327-0

**Published:** 2021-07-14

**Authors:** Rudolph Fredua-Agyeman, Sheau-Fang Hwang, Hui Zhang, Igor Falak, Xiuqiang Huang, Stephen E. Strelkov

**Affiliations:** 1 Department of Agricultural, Food and Nutritional Science, University of Alberta, Edmonton, AB T6G 2P5 Canada; 2 Institute of Vegetables and Flower, Chinese Academy of Agricultural Sciences, Beijing, 100081 China; 3 Georgetown (Caledon) Research Centre, Corteva Agriscience, 12111 Mississauga Rd, Caledon, ON L7C 1X1 Canada

**Keywords:** Plant sciences, Plant breeding, Plant genetics

## Abstract

In this study, clubroot resistance in the resynthesized European winter *Brassica napus* cv. ‘Tosca’ was introgressed into a Canadian spring canola line ‘11SR0099’, which was then crossed with the clubroot susceptible spring line ‘12DH0001’ to produce F_1_ seeds. The F_1_ plants were used to develop a doubled haploid (DH) mapping population. The parents and the DH lines were screened against ‘old’ pathotypes 2F, 3H, 5I, 6M and 8N of the clubroot pathogen, *Plasmodiophora brassicae*, as well as against the ‘new’ pathotypes 5X, 5L, 2B, 3A, 3D, 5G, 8E, 5C, 8J, 5K, 3O and 8P. Genotyping was conducted using a *Brassica* 15K SNP array. The clubroot screening showed that ‘Tosca, ‘11SR0099’ and the resistant DH lines were resistant to three (2F, 3H and 5I) of the five ‘old’ pathotypes and four (2B, 3O, 8E and 8P) of the 12 ‘new’ pathotypes, while being moderately resistant to the ‘old’ pathotype 8N and the ‘new’ pathotypes 3D and 5G. ‘Tosca’ was susceptible to isolates representing pathotype 3A (the most common among the ‘new’ pathotypes) as well as pathotypes 6M, 5X, 5L, 5K and 8J. Linkage analysis and QTL mapping identified a ca. 0.88–0.95 Mb genomic region on the A03 chromosome of ‘Tosca’ as conferring resistance to pathotypes 2F, 3H, 5I, 2B, 3D, 5G, 8E, 3O and 8P. The identified QTL genomic region housed the *CRk*, *Crr3* and *CRd* gene(s). However, the susceptibility of ‘Tosca’ to most of the common virulent pathotypes makes it unattractive as a sole CR donor in the breeding of commercial canola varieties in western Canada.

## Introduction

Rapeseed (*Brassica napus* and *B. rapa*) was the second most important oilseed crop (71.9 MMT) after soybeans (362.0 MMT) worldwide in 2018–2019^1^. The leading producers include Canada, the European Union, China, India, Australia, Russia and the United Kingdom (FAOSTAT). In Canada, rapeseed varieties (including *B*. *juncea*) that contain < 2% erucic acid and < 30 µmol glucosinolate per gram of air-dried oil-free meal are referred to as canola, derived from “Canadian oil”^2,3^. According to the Canola Council of Canada, approximately 8.6 million ha of canola were planted in 2019, yielding 19.6 MMT of seed or 28% of the world’s production^4^. The Prairie Provinces, namely Alberta, Saskatchewan and Manitoba, accounted for 28%, 55% and 16%, respectively, of total canola produced in Canada in 2019^4^. The continued profitability of canola has led to monocropping of canola in many regions of the Prairies and, globally, rapeseed production has increased by 12% increase over the past 10 years (FAOSTAT).

Unfortunately, the intensified cultivation of *Brassica* crops worldwide has led to an increased incidence and severity of many diseases and the emergence of virulent isolates of the causal organisms^5–10^. In Canada, clubroot caused by the obligate parasite *Plasmodiophora brassicae*, has become a major threat to canola production due its spread across Alberta^11^ and into Saskatchewan and Manitoba^12,13^. The disease is managed primarily by the planting of clubroot resistant cultivars, which have allowed the continued cultivation of canola even in fields that are heavily infested by *P. brassicae*^14^. In recent years, however, ‘new’ virulent pathotypes of *P. brassicae* capable of overcoming this resistance have emerged in Alberta^8,15,16^; in Saskatchewan and Manitoba, most isolates are still avirulent on clubroot resistant canola cultivars, although a virulent pathotype was recently confirmed in Manitoba^16^.

Clubroot resistance in Canadian canola varieties was derived from the European winter *B. napus* cultivar ‘Mendel’^17,18^. Since most current commercial canola varieties do not possess resistance to isolates representing the ‘new’ *P. brassicae* pathotypes, there is a need to identify and utilize additional resistance sources for development of the next generation of clubroot resistant cultivars. This task is especially daunting in Alberta, where various novel pathotypes have become widespread^8,15,16^. In this study, clubroot resistance (CR) derived from the resynthesized Swedish winter *Brassica napus* cv. ‘Tosca’^19^ was evaluated against 18 isolates representing ‘old’ and ‘new’ pathotypes of *P. brassicae* from Alberta. ‘Tosca’ was developed through many breeding cycles and hence is a stable cultivar. To understand the genetic basis of the resistance, ‘Tosca’ was used in genetic crosses with a Canadian spring canola line to develop a clubroot resistant spring-type canola. A doubled haploid mapping population developed from F_1_ plants of the clubroot resistant spring line and a clubroot susceptible Canadian spring canola line was genotyped with a *Brassica* 15K SNP array, and linkage analysis and QTL mapping were conducted to identify genomic regions associated with clubroot resistance from ‘Tosca’.

## Results

### Clubroot assessment in ECD04, ECD05, ‘Westar’ and ‘Tosca’

The results of the inoculation experiments (Table 1) showed that the resistant check, *B. rapa* subsp. *rapifera* ECD 04, was completely resistant (mean ID 0.00% ± 0.00%) to all 18 *P. brassicae* isolates (representing 17 unique pathotypes). In contrast, the susceptible checks, *B. rapa* var. *pekinensis* ECD 05 and the *B. napus* cv. ‘Westar’, were susceptible to all isolates (mean ID ranged from 96.88% ± 0.53% to 100.0% ± 0.0% and from 96.08% ± 0.47% to 100.0% ± 0.0%, respectively) (Table 1). The *B*. *napus* cv. ‘Tosca’ included as a check in the inoculation experiments was resistant to pathotypes 2F (6.94% ± 2.41%), 3H (6.67% ± 2.13%), 5I (11.76% ± 3.05%), 2B (10.61% ± 1.54%), 8E (15.94% ± 0.62%), 3O (12.50% ± 2.77%) and 8P (1.75% ± 0.64%), moderately resistant to pathotypes 8N (37.68% ± 1.62%), 3D (42.53% ± 0.73%) and 5G (33.33% ± 1.22%), and susceptible to pathotypes 6M (50.79% ± 2.78%), 5X (L-G1 and L-G2; 90.48% ± 1.58% and 94.20% ± 0.95%, respectively), 5L (98.33% ± 0.63%), 3A (82.61% ± 2.50%), 5C (48.15% ± 3.30%), 5K (82.72% ± 1.14%) and 8J (90.74% ± 2.27%) (Table 1).

**Table 1:** Clubroot severity data for ‘11SR0099’ (clubroot-resistant doubled haploid (DH) parent), ‘12DH0001’ (clubroot-susceptible DH parent), DH individual lines and population, *B. napus* cv. ‘Tosca’, *B. rapa* subsp. *rapifera* European Clubroot Differential (ECD) 04, *B. rapa* var. *pekinensis* ECD 05, and the *B. napus* cv. ‘Westar’.

Pathotype (isolate)	CR DH parent ‘11SR0099’ Mean ± SEM	CS DH parent ‘12DH0001’ Mean ± SEM	Individual DH lines		DH population	*B. napus* cv. ‘Tosca’ Mean ± SEM	*B. rapa* ‘ECD 04’ Mean ± SEM	*B. rapa* ‘ECD 05’ Mean ± SEM	*B. napus* cv. ‘Westar’ Mean ± SEM
			Minimum	Maximum	(118 lines)				
			Mean ± SEM	Mean ± SEM	Mean ± SEM				
2F	12.63 ± 1.34^a^	96.07 ± 3.08^a^	5.25 ± 0.71	100.00 ± 0.00	71.31 ± 1.77	6.94 ± 2.41	0.00 ± 0.00	98.96 ± 0.33	100.00 ± 0.00
3H	8.25 ± 2.27^a^	98.83 ± 1.17^a^	0.63 ± 0.31	98.37 ± 0.88	58.97 ± 2.17	6.67 ± 2.13	0.00 ± 0.00	98.72 ± 0.47	98.61 ± 0.43
5I	22.17 ± 2.69^a^	99.53 ± 0.47^a^	9.09 ± 4.77	99.07 ± 0.53	75.28 ± 1.64	11.76 ± 3.05	0.00 ± 0.00	99.02 ± 0.48	100.00 ± 0.00
6M	91.67 ± 4.41^b^	93.33 ± 6.67	22.53 ± 8.11	99.01 ± 0.54	91.87 ± 0.66	50.79 ± 2.78^b^	0.00 ± 0.00	100.00 ± 0.00	100.00 ± 0.00
8N	39.97 ± 3.58^a^	100 ± 0.00^a^	0.00 ± 0.00	100.00 ± 0.00	88.38 ± 1.34	37.68 ± 1.62	0.00 ± 0.00	96.88 ± 0.53	96.08 ± 0.47
5X (L-G1)	73.82 ± 7.15^a^	100 ± 0.00^a^	33.33 ± 5.81	100.00 ± 0.00	93.14 ± 0.96	90.48 ± 1.58	0.00 ± 0.00	98.72 ± 0.47	100.00 ± 0.00
5X (L-G2)	83.33 ± 3.33^ab^	100 ± 0.00^a^	25.00 ± 6.78	100.00 ± 0.00	88.84 ± 1.07	94.20 ± 0.95^b^	0.00 ± 0.00	97.53 ± 0.64	100.00 ± 0.00
5L	93.33 ± 6.67	99.56 ± 0.44	33.33 ± 10.40	100.00 ± 0.00	94.37 ± 0.75	98.33 ± 0.63	0.00 ± 0.00	98.04 ± 0.81	96.30 ± 1.60
2B	2.80 ± 2.80^a^	100.00 ± 0.00^a^	0.00 ± 0.00	100.00 ± 0.00	65.91 ± 2.33	10.61 ± 1.54	0.00 ± 0.00	97.53 ± 1.07	97.92 ± 0.98
3A	72.22 ± 9.09^a^	99.42 ± 0.58^a^	0.00 ± 0.00	100.00 ± 0.00	89.63 ± 1.51	82.61 ± 2.50	0.00 ± 0.00	100.00 ± 0.00	100.00 ± 0.00
3D	46.83 ± 2.38^a^	99.17 ± 0.83^a^	0.00 ± 0.00	100.00 ± 0.00	70.93 ± 2.06	42.53 ± 0.73	0.00 ± 0.00	96.97 ± 1.42	97.62 ± 0.49
5C	45.25 ± 2.90^a^	96.67 ± 3.33^a^	0.00 ± 0.00	100.00 ± 0.00	86.67 ± 1.64	48.15 ± 3.30	0.00 ± 0.00	97.10 ± 0.87	100.00 ± 0.00
5G	43.61 ± 5.64^a^	99.02 ± 0.94^a^	0.00 ± 0.00	100.00 ± 0.00	78.23 ± 1.73	33.33 ± 1.22	0.00 ± 0.00	100.00 ± 0.00	100.00 ± 0.00
8E	11.94 ± 1.94^a^	100 ± 0.00^a^	0.00 ± 0.00	100.00 ± 0.00	64.26 ± 2.38	15.94 ± 0.62	0.00 ± 0.00	100.00 ± 0.00	96.97 ± 0.82
5K	86.31 ± 2.94^a^	99.25 ± 0.75^a^	8.33 ± 4.45	100.00 ± 0.00	94.44 ± 0.89	82.72 ± 1.14	0.00 ± 0.00	97.44 ± 0.46	100.00 ± 0.00
8J	71.11 ± 4.44^ab^	99.45 ± 0.55^a^	20.00 ± 5.85	100.00 ± 0.00	94.52 ± 0.91	90.74 ± 2.27^b^	0.00 ± 0.00	100.00 ± 0.00	96.30 ± 0.88
3O	8.89 ± 1.15^a^	97.47 ± 2.36^a^	0.00 ± 0.00	100.00 ± 0.00	65.90 ± 2.23	12.50 ± 2.77	0.00 ± 0.00	100.00 ± 0.00	98.92 ± 0.50
8P	10.44 ± 1.38^ab^	97.25 ± 1.66^a^	0.00 ± 0.00	100.00 ± 0.00	62.88 ± 2.3	1.75 ± 0.64^b^	0.00 ± 0.00	98.29 ± 0.31	96.19 ± 0.74

^a^Clubroot severity (index of disease, ID %) was significantly (*P* < 0.05) different in the resistant DH parent (‘11SR0099’) compared to the susceptible DH parent (‘12DH0001’).

^b^Clubroot severity was significantly (*P* < 0.05) different in the *Brassica napus* cv. ‘Tosca’ compared to the resistant DH parent (‘11SR0099’).

### Clubroot assessment in DH parents and DH population

There was a significant difference (*p* < 0.05) between the mean ID values of the clubroot resistant DH parent ‘11SR0099’ and the clubroot susceptible DH parent ‘12DH0001’ in 16 of the 18 *P. brassicae* isolates, with the exception of pathotypes 6 M and 5L (Table 1). The DH parent ‘11SR0099’ was resistant to *P. brassicae* pathotypes 2F (12.63% ± 1.34%), 3H (8.25% ± 2.27%), 5I (22.17% ± 2.69%), 2B (2.80% ± 2.80%), 8E (11.94% ± 1.94%), 3O (8.89% ± 1.15%) and 8P (10.44% ± 1.38%). It was moderately resistant to pathotypes 8N (39.97% ± 3.58%), 3D (46.83% ± 2.38%), 5G (43.61% ± 5.64%) and 5C (45.25% ± 2.90%), and susceptible to pathotypes 6M (91.67% ± 4.41%), 5X (L-G1 and L-G2; 73.82% ± 7.15% and 83.33% ± 3.33%, respectively), 5L (93.33% ± 6.67%), 3A (72.22% ± 9.09%), 5K (86.31% ± 2.94%) and 8J (71.11% ± 4.44%) (Table 1). The difference in the mean ID values between the DH parent ‘11SR0099’ and the *B. napus* cv. ‘Tosca’ was not significant for pathotypes 2F, 3H, 5I, 8N, 5X (L-G1), 5L, 2B, 3A, 3D, 5C, 5G, 8E, 5K and 3O (i.e. except pathotypes 6M, 5X (L-G2), 8J and 8P) (Table 1). Therefore, the disease reactions of the clubroot resistant DH parent ‘11SR0099’ followed a similar pattern as ‘Tosca’. In contrast, the DH parent ‘12DH0001’ was susceptible to all 18 isolates (mean ID values in the range of 93.33% ± 6.67% to 100.00% ± 0.00%, Table 1).

**Figure 1:**
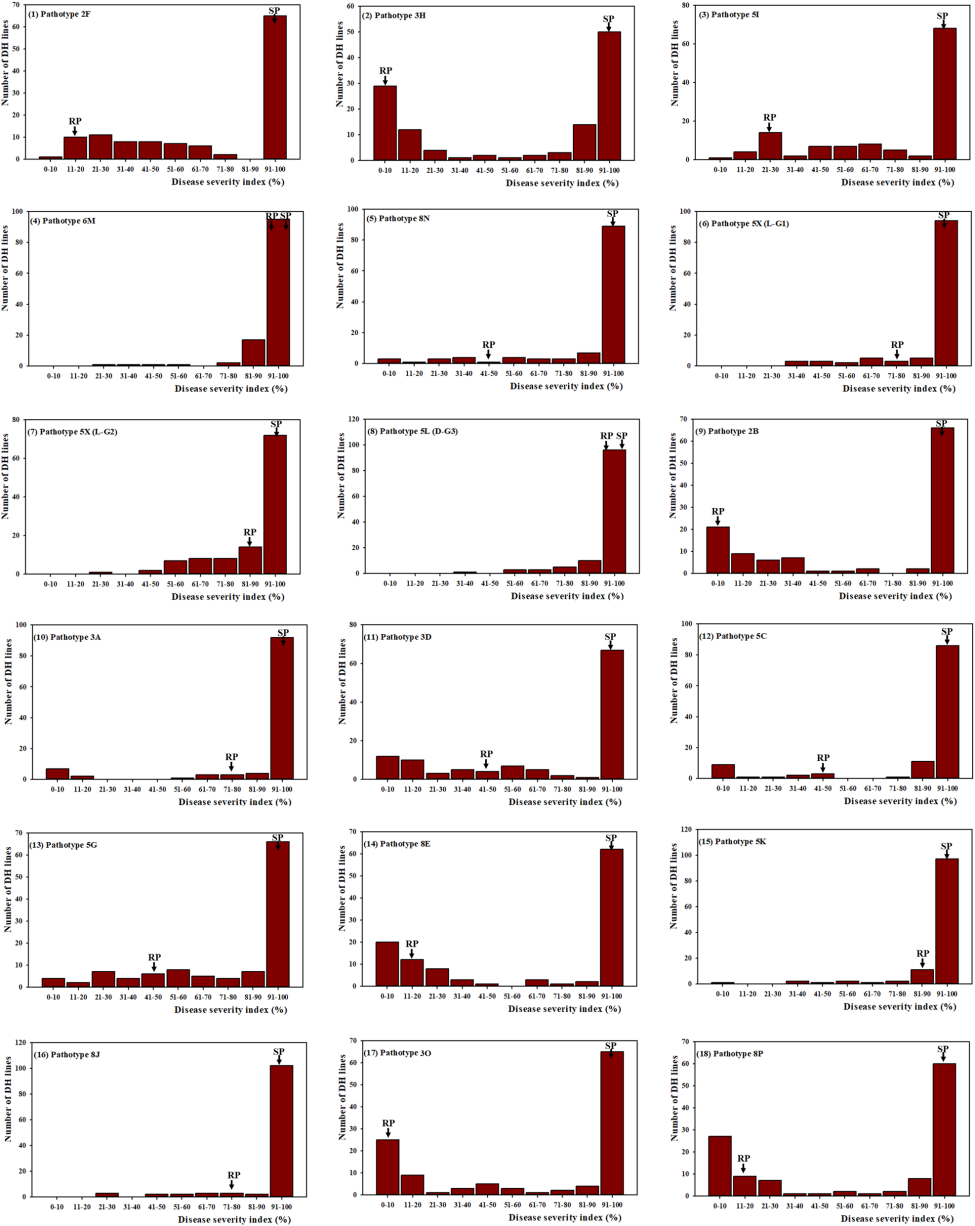
Frequency distribution of doubled haploid lines derived from the *Brassica napus* cv. ‘Tosca’ for resistance to *Plasmodiophora brassicae* single-spore isolates (SSIs) representing pathotypes 2F (1), 3H (2), 5I (3), 6M (4) and 8N (5), and field isolates representing pathotypes 5X (L-G1(6) and L-G2(7)), 5L (8), 2B (9), 3A (10), 3D (11), 5C (12), 5G (13), 8E (14), 5K (15), 8J (16), 3O (17) and 8P (18). The SSIs and field isolates were identified prior to and after the introduction of clubroot-resistant canola cultivars in Canada, respectively.

Clubroot severity in the replicated greenhouse experiments was significantly correlated and ranged from *r* = 0.60 to 0.98, *p* < 0.0001 (Table S1). In addition, a significant genotypic effect (*p* < 0.05) and high heritability (57.68% to 99.94%) were detected in the DH population (Table S1). Thus, the combined data from the individual experiments is presented. Figure 1 shows the frequency distribution of the combined clubroot severity for the 116 DH lines inoculated with 18 *P. brassicae* isolates. The Shapiro–Wilk test suggested that none of the data followed a normal distribution, but rather the distributions skewed mostly to the left (Fig. 1). The mean ID ± SEM of the DH lines ranged from 58.97% ± 2.17% to 94.52% ± 0.91% (Table 1). Despite the high mean ID values across all isolates, segregation of clubroot resistance in the DH population was apparent (19.8% to 40.5% R and MR) in the greenhouse experiments carried out with nine (2F, 3H, 5I, 2B, 3D, 5G, 8E, 3O and 8P) of the 17 *P. brassicae* pathotypes. Segregation for clubroot resistance in the DH population for the remaining eight (6M, 8N, 5X (L-G1 and L-G2), 5L, 3A, 5C, 5K and 8J) pathotypes (represented by nine isolates) was very small (0.9% to 13.4% R and MR). Chi-square goodness of fit tests showed that segregation for clubroot resistance in the DH population inoculated with all 18 isolates was significantly different (*p* < 0.05) from the expected 1:1 segregation ratio (Table 2). In comparison, Chi-square tests at *p* < 0.01 fit the hypothesized 1:1 segregation ratio for the DH population inoculated with pathotypes 3H, 2B and 8E, and marginally for pathotype 8P (Table 2).

**Table 2:** Chi-square tests for 1:1 segregation ratio for doubled haploid (DH) lines produced from F_1_ plants obtained from the cross ‘11–99’ (*Brassica napus* cv. ‘Tosca’ (clubroot resistant) × ‘12–1’ (clubroot susceptible)) screened for resistance to 18 *Plasmodiophora brassicae* isolates under greenhouse conditions.

*P. brassicae *pathotypes	Number of individuals			Test of 1:1 ratio (R + MR: S)	
	ID 0–30% (R)	ID 31–50% (MR)	ID 51–100% (S)	χ^2^	Probability
2F	21	16	79	15.2	9.64E−05
3H	44	3	69	4.2	0.0411
5I	18	9	89	33.1	8.58E−09
6M	1	2	113	104.3	1.73E−24
8N	7	4	105	76.2	2.60E−18
5X (L-G1)	0	6	107	90.3	2.07E−21
5X (L-G2)	1	2	107	98.3	3.55E−23
5L (D-G3)	0	1	115	112.0	3.51E−26
2B	35	8	70	6.5	0.0111
3A	9	0	101	76.9	1.76E−18
3D	24	9	81	20.2	6.94E−06
5C	11	4	97	60.0	9.32E−15
5G	12	10	89	40.4	2.03E−10
8E	39	4	67	5.2	0.0221
5K	1	3	111	99.6	1.91E−23
8J	3	2	110	95.9	1.23E−22
3O	34	8	74	8.8	0.0030
8P	42	2	72	6.8	0.0093

*Plasmodiophora brassicae* pathotype designations are based on the Canadian Clubroot Differential (CCD) set^15^. Pathotypes 2F, 3H, 5I, 6M and 8N are single-spore isolates collected prior to the introduction of clubroot resistant canola (Strelkov et al.^20^; Xue et al. ^21^). Pathotypes 5X (L-G1 and L-G2) and 5L are field isolates collected in 2013 (Strelkov et al.^18,20^). Pathotypes 2B, 3A, 3D, 5C, 5G and 8E are field isolates collected in 2014 (Strelkov et al. ^20,22^). Pathotypes 5K and 8J are field isolates collected in 2015 (Strelkov et al. ^20,23^). Pathotypes 3O and 8P are field isolates collected in 2016 (Strelkov et al. ^20,24^).

### Genetic linkage mapping

The initial filtering steps removed 10,437 (76.1%) of the 13,714 SNP markers. These comprised 445 (3.2%) SNP markers which failed to amplify genomic DNA in the parents, 492 (3.6%) SNP markers that were monomorphic in the parents, 2149 (15.7%) SNP markers that were monomorphic in the DH population, and 7351 (53.6%) markers that had minor-allele frequency ≤ 5% and were missing data points for > 5% in the DH population. Chi-square tests on the remaining 3277 (23.9%) SNP markers showed that 2365 (17.3%) SNP markers fit the 1:1 segregation ratio expected for a DH population (*p* < 0.05), 785 (5.7%) of the markers showed ‘minor’ segregation distortion (*p* < 1.67 × 10^–5^), and 127 (0.9%) were highly distorted and hence could be discarded. Therefore, only 23.0% of the initial markers used for screening the DH population were used for linkage map construction.

Linkage analysis distributed 2253 of the 2365 markers, which fit a 1:1 Mendelian ratio expected for a DH population on 24 linkage groups (Table S3). Markers on 14 of the 24 linkage groups corresponded to markers on 14 (A01, A02, A04, A05, A06, A07, A08, A10, C02, C03, C04, C05, C07 and C09) of the 19 chromosomes of *B*. *napus*. Markers on two linkage groups each corresponded to markers on chromosomes A09 and C01, while markers on three linkage groups each corresponded to markers on chromosomes A03 and C08 of *B*. *napus*. A parallel linkage analysis distributed 2969 of the 3150 (2365 + 785) ‘Mendelian’ and ‘distorted’ SNP markers on 20 linkage groups (Table 3). Markers on 16 of the 20 linkage groups corresponded to markers on 16 (A01, A02, A03, A04, A05, A06, A07, A08, A10, C01, C02, C03, C04, C05, C07 and C09) chromosomes of *B*. *napus*. Two linkage groups each represented chromosomes A09 and C08. Linkage analyses did not place any of the filtered markers on chromosome C06 (Tables S3 and 3).

**Table 3:** The distribution of 2969 ‘Mendelian’ and ‘distorted’ SNP markers on 20 linkage groups representing 18 of the 19 chromosomes used to map QTL for clubroot resistance in doubled haploid lines derived from the *Brassica napus* cv. ‘Tosca’.

Chromosome	Linkage group	Number of SNP markers used for QTL mapping		Total map length/(cM)	Marker density/cM
		Actual	Bin^a^		
A01	1	245	83	134.4	1.8
A02	2	71	32	62.1	1.1
A03	3	231	101	182.4	1.3
A04	4	185	90	163.9	1.1
A05	5	20	8	14.2	1.4
A06	6	289	102	188.2	1.5
A07	7	204	53	77.9	2.6
A08	8	248	65	127.4	1.9
A09a	9	56	19	77.1	0.7
A09b	10	70	39	66.8	1.0
A10	11	183	56	100.9	1.8
C01	12	152	27	57.7	2.6
C02	13	153	39	169.5	0.9
C03	14	232	57	156.6	1.5
C04	15	31	10	20.6	1.5
C05	16	143	63	150.4	1.0
C07	17	232	77	184.4	1.3
C08a	18	122	33	72.7	1.7
C08b	19	31	12	24.3	1.3
C09	20	71	34	83.3	0.9
Total or average		2969	1000	2114.8	1.4

^a^Multiple markers that mapped to the same position on the linkage map were put in the same bin.

Using only the ‘Mendelian’ markers, the linkage group lengths ranged from 13.1 cM (linkage group 4) to 189.4 cM (linkage group 20), while the total length was 2211.5 cM (Table S3). The number of ‘Mendelian’ markers per chromosome ranged from 5 to 204 and averaged 123.5 markers, while the marker density per cM ranged from 0.1 to 2.7 and averaged 1.1 markers per cM (Table S3). In the case of the use of the ‘Mendelian’ and ‘distorted’ markers, the linkage group lengths ranged from 14.2 cM (linkage group 5) to 188.2 cM (linkage group 6), while the total length was 2114.8 cM (Table 3). The number of ‘Mendelian’ and ‘distorted’ markers per chromosome ranged from 20 to 289 and averaged 164.9 markers, whereas the marker density per cM ranged from 0.7 to 2.6 and averaged 1.4 markers per cM (Table 3).

### Additive-effect QTL analysis

QTL analysis conducted by the CIM method with 829 Bin ‘Mendelian’ markers (Table S4) detected 15 coincident QTL on chromosome A03, which were significantly associated with resistance to *P. brassicae* pathotypes 2F, 3H, 5I, 2B, 3D, 5G, 8E, 3O and 8P. Based on the *R*^2^ values, five of the QTL were major-effect QTL, nine were moderate-effect QTL and one was a minor-effect QTL. The peak LOD values for the QTL ranged from 6.8 to 48.1 (Table S4). The SNP markers Bn_A03_p14784764 and Bn_A03_p15704830, which were within two-LOD confidence intervals and spanned 20.6 cM (at position 56.6 to 77.2 cM), bordered the genomic region conferring resistance to the nine *P. brassicae* pathotypes according to the use of the ‘Mendelian’ markers (Fig. 3).

**Figure 2:**
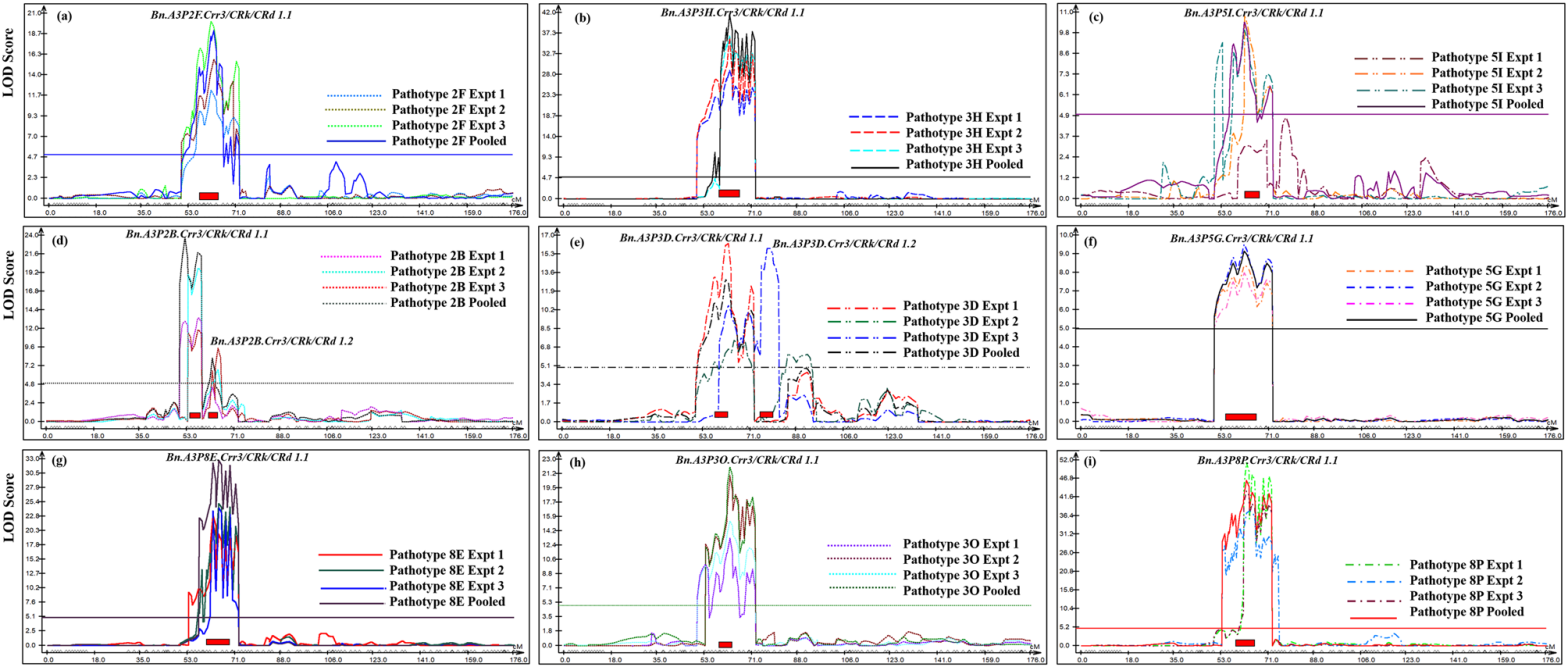
QTL likelihood profile of the A03 chromosome of *Brassica napus* obtained by use of both ‘Mendelian’ and low ‘distorted’ markers. The peak regions indicate genomic regions conferring resistance to nine *Plasmodiophora brassicae* pathotypes, 2F (**a**), 3H (**b**), 5I (**c**), 2B (**d**), 3D (**e**), 5G (**f**), 8E (**g**), 3O (**h**) and 8P (**i**). Clubroot resistance in the DH lines was derived from the *Brassica napus* cv. ‘Tosca’. The LOD scores are indicated on the y-axis and the QTL names positioned at the peak of each profile.

**Figure 3:**
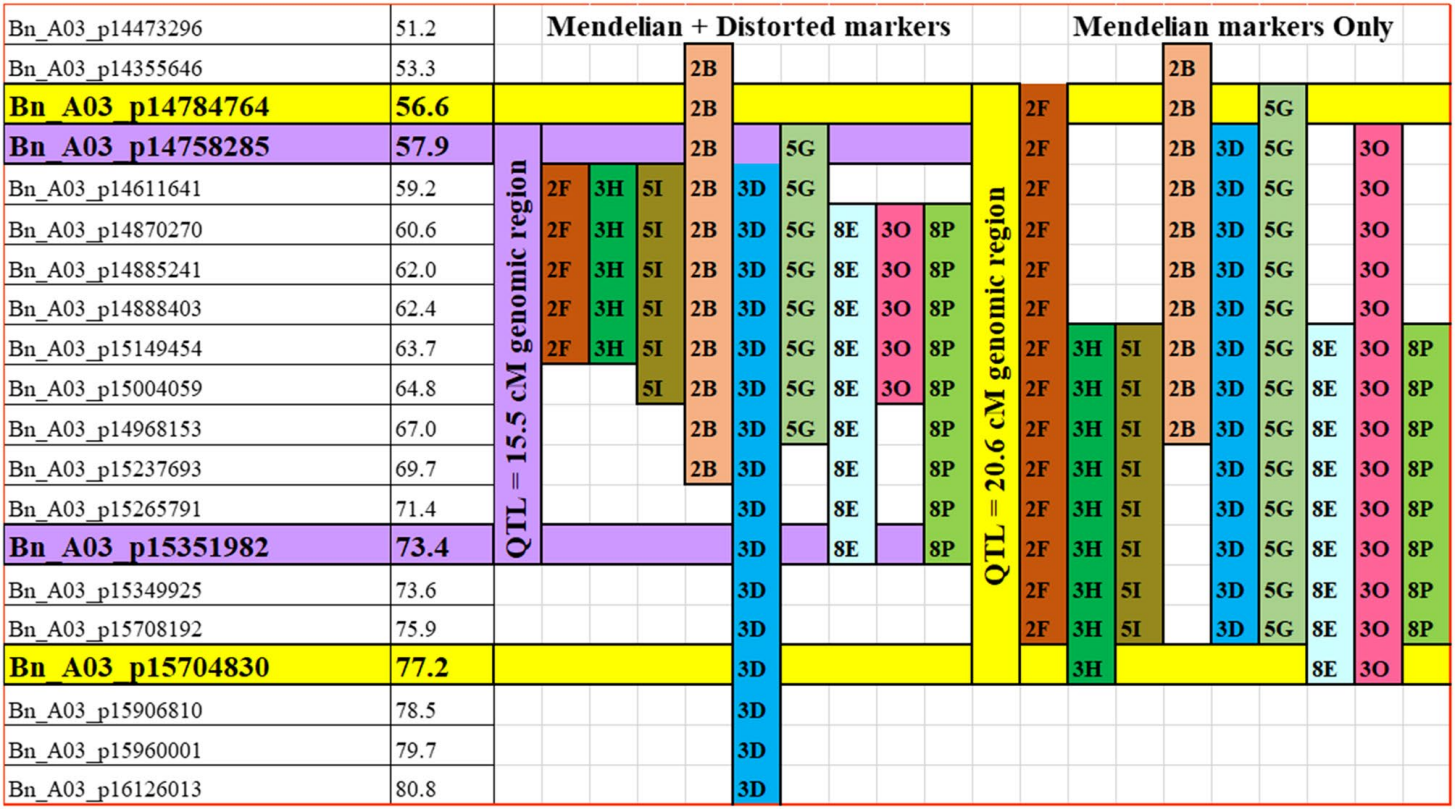
Single-nucleotide polymorphism (SNP) markers and different-sized genomic fragments on the 24.3–26.1 Mb region of the *Brassica napus* genome (ca. 14.7–15.7 Mb on the *Brassica rapa* genome), conferring resistance to *Plasmodiophora brassicae* pathotypes 2F, 3H, 5I, 2B, 3D, 5G, 8E, 3O and 8P.

QTL analysis conducted with the 1000 ‘Mendelian’ and ‘distorted’ markers detected 11 coincident QTL, which were associated with resistance to pathotypes 2F, 3H, 5I, 2B, 3D, 5G, 8E, 3O and 8P (Table 4). The QTL profiles with the ‘Mendelian’ and ‘distorted’ markers (Fig. 2) were similar to those obtained by use of the ‘Mendelian’ markers (Fig. S1). The SNP markers Bn_A03_p14758285 (57.9 cM) and Bn_A03_p15351982 (73.4 cM) spanned the major QTL identified by the use of the ‘Mendelian’ and ‘distorted’ markers (Fig. 3). Based on the *R*^2^ values, four of the QTL were major-effect QTL, five were moderate-effect QTL and two were minor-effect QTL. The peak LOD values ranged from 6.7 to 51.2 and the two-LOD confidence interval spanned 15.5 cM (Fig. 3).

**Table 4:** Summary of QTL on chromosome A03 associated with clubroot resistance in doubled haploid lines derived from the *Brassica napus* cv. ‘Tosca’ inoculated with different *Plasmodiophora brassicae* pathotypes using ‘Mendelian’ and ‘distorted’ markers.

Identified QTL	Pathotype	Expt	QTL pisitions (cM)^a^		Left SNP Marker	Right SNP marker	LOD	Additive	R^2^ (%)
			Peak	Conf Interval					
*Bn.A3P2F.Crr3/CRk/CRd 1.1*	2F	1	61.5	60.4–63.6	Bn_A03_p14583041	Bn_A03_p15149454	12.2	− 30.8	17.1
		2	62.5	61.1–63.6	Bn_A03_p14870270	Bn_A03_p15149454	15.9	− 26.3	18.6
		3	61.5	60.4–63.3	Bn_A03_p14583041	Bn_A03_p15149454	20.1	− 31.3	34.5
		Pooled	62.5	61.5–63.5	Bn_A03_p14870270	Bn_A03_p15149454	19.2	− 29.5	37.2
*Bn.A3P3H.Crr3/CRk/CRd 1.1*	3H	1	62.5	61.6–63.6	Bn_A03_p14870270	Bn_A03_p15149454	29.0	− 33.8	49.9
		2	62.5	61.5–62.8	Bn_A03_p14870270	Bn_A03_p15149454	36.7	− 38.7	93.3
		3	62.5	61.7–63.1	Bn_A03_p14870270	Bn_A03_p15149454	36.8	− 39.4	90.6
		Pooled	62.5	62.2–63.1	Bn_A03_p14885241	Bn_A03_p15149454	41.6	− 37.2	92.5
*Bn.A3P5I.Crr3/CRk/CRd 1.1*	5I	2	61.5	61.4–64.6	Bn_A03_p14870270	Bn_A03_p15004059	10.7	− 27.8	27.7
		3	61.5	59.8–63.7	Bn_A03_p14583041	Bn_A03_p15004059	10.0	− 28.2	19.3
		Pooled	61.5	60.2–63.3	Bn_A03_p14583041	Bn_A03_p15149454	10.4	− 25.9	18.3
*Bn.A3P2B.Crr3/CRk/CRd 1.1*	2B	1	57.4	55.8–58.4	Bn_A03_p14355646	Bn_A03_p14611641	13.3	− 33.7	29.6
		2	57.4	55.8–58.4	Bn_A03_p14355646	Bn_A03_p14611641	19.6	− 37.2	54.6
		3	57.4	55.7–58.4	Bn_A03_p14355646	Bn_A03_p14611641	11.8	− 31.9	26.1
		Pooled	57.4	53.8–58.4	Bn_A03_p14355646	Bn_A03_p14611641	23.4	− 41.9	72.7
*Bn.A3P2B.Crr3/CRk/CRd 1.2*	2B	2	64.7	62.2–67.8	Bn_A03_p14885241	Bn_A03_p15237693	6.7	− 40.4	19.4
		3	64.7	63.7–65.7	Bn_A03_p14888403	Bn_A03_p14968153	9.3	− 42.5	15.6
		Pooled	62.5	61.5–64.6	Bn_A03_p14870270	Bn_A03_p15004059	8.2	− 41.5	15.5
*Bn.A3P3D.Crr3/CRk/CRd 1.1*	3D	1	62.5	60.5–65.7	Bn_A03_p14583041	Bn_A03_p14968153	16.2	− 30.3	23.2
		2	64.7	63.7–66.3	Bn_A03_p14888403	Bn_A03_p14968153	7.5	− 20.4	11.5
		3	62.5	60.5–65.2	Bn_A03_p14583041	Bn_A03_p14968153	10.4	− 24.8	16.0
		Pooled	61.5	60.3–63.4	Bn_A03_p14583041	Bn_A03_p15149454	13.0	− 25.6	15.8
*Bn.A3P3D.Crr3/CRk/CRd 1.2*	3D	3	77.2	75.9–80.6	Bn_A03_p15704830	Bn_A03_p16126013	15.8	− 31.4	36.4
*Bn.A3P5G.Crr3/CRk/CRd 1.1*	5G	1	61.7	58.7–65.7	Bn_A03_p14758285	Bn_A03_p14968153	8.3	− 20.6	34.2
		2	61.7	58.7–65.7	Bn_A03_p14758285	Bn_A03_p14968153	9.5	− 20.7	37.8
		3	61.7	58.7–66.3	Bn_A03_p14758285	Bn_A03_p14968153	8.0	− 21.6	32.4
		Pooled	61.7	58.7–66.3	Bn_A03_p14758285	Bn_A03_p14968153	9.0	− 20.1	36.7
*Bn.A3P8E.Crr3/CRk/CRd 1.1*	8E	1	62.5	61.5–72.0	Bn_A03_p14870270	Bn_A03_p15351982	23.3	− 41.1	74.1
		2	64.7	64.4–69.1	Bn_A03_p14927037	Bn_A03_p15237693	25.2	− 40.5	67.6
		3	64.7	64.5–69.7	Bn_A03_p14927037	Bn_A03_p15265791	24.3	− 42.2	84.5
		Pooled	64.7	64.4–69.2	Bn_A03_p14927037	Bn_A03_p15237693	32.8	− 41.3	90.5
*Bn.A3P3O.Crr3/CRk/CRd 1.1*	3O	1	62.5	61.2–63.5	Bn_A03_p14870270	Bn_A03_p15149454	13.3	− 40.5	19.8
		2	62.5	61.5–63.5	Bn_A03_p14870270	Bn_A03_p15149454	21.0	− 37.1	35.1
		3	62.5	61.4–64.2	Bn_A03_p14870270	Bn_A03_p15004059	15.5	− 31.6	23.2
		Pooled	62.5	61.5–63.6	Bn_A03_p14870270	Bn_A03_p15149454	22.2	− 37.3	31.7
*Bn.A3P8P.Crr3/CRk/CRd 1.1*	8P	1	62.5	61.5–63.5	Bn_A03_p14870270	Bn_A03_p15149454	51.2	− 43.2	95.0
		2	63.7	62.2–65.7	Bn_A03_p14885241	Bn_A03_p14968153	38.0	− 39.3	46.1
		3	62.5	61.5–63.5	Bn_A03_p14870270	Bn_A03_p15149454	43.1	− 40.8	90.2
		Pooled	62.5	61.5–63.5	Bn_A03_p14870270	Bn_A03_p15149454	46.4	− 41.5	92.7

^a^QTL positions based on two-LOD support intervals for 99% confidence interval (CI), (Lander and Botstein^52^).

Overall, the use of the ‘Mendelian’ markers and both the ‘Mendelian’ and ‘distorted’ markers for the analysis yielded comparable LOD and *R*^2^ values, as well as coincident QTL for resistance to nine (2F, 3H, 5I, 2B, 3D, 5G, 8E, 3O and 8P) of the 18 isolates used to screen the DH lines. No QTL were detected for resistance to *P*. *brassicae* pathotypes 6M, 8N, 5X (L-G1 and L-G2), 5L, 3A, 5C, 8J or 5K. Furthermore, the additive effect of the QTL detected in both cases had negative values (Tables 4 and S4). This indicated that the favorable allele for resistance originated from ‘Tosca’.

### QTL genomic region

The 15.5 to 20.6 cM genomic region flanking the QTL region represented an 837.6 Kb (LK031800 at positions 887,529 to 1,725,145b) region on the *B*. *napus* genome and a 946.6 Kb (A03 chromosome at positions 14,757,826 to15,704,427 b) region on the of the *B. rapa* genome (Table 5). The SNP markers in this region matched GenBank entries corresponding to proteasome family proteins, Calcium-dependent lipid-binding family proteins, zinc finger containing proteins, multisubstrate pseudouridine synthase, serine/threonine-protein kinase WNK1 and RBK2, HCO3-transporter family proteins, alpha/beta-Hydrolases superfamily proteins, E3 ubiquitin ligase, peptidyl-prolyl isomerase, putative DNA repair protein, electron transport SCO1/SenC family proteins and transcriptional factor B3 family proteins (Table 5). Moreover, the QTL region contained six (LOC103860116, LOC103859010, LOC103859018, LOC103859177, LOC103859225 and LOC103859386) leucine-rich repeat (LRR) kinases, which have been identified as disease resistance-related genes (Table 6).

**Table 5:** Description of the single-nucleotide polymorphism (SNP) markers flanking the major QTL associated with resistance to *Plasmodiophora brassicae* pathotypes 2F, 3H, 5I, 2B, 3D, 5G, 8E, 3O and 8P.

SNP marker	Overlapping gene(s)	Species	Genomic location	SNP marker position		Expect (E)-value	Description of gene functions
				Start	End		
Bn_A03_p14473296	BnaA03g27790D	*B. napus*	LK031800	2009415	2009519	3.2e−50	Proteasome family protein
	Bra001036	*B. rapa*	A03	14472892	14472996	2.9e−48	
Bn_A03_p14355646	BnaA03g27480D	*B. napus*	LK031800	2118648	2118712	7.6e−26	Uncharacterized protein Mb2253c-like
	LOC103858911	*B. rapa*	A03	14773527	14773591	2e−21	
Bn_A03_p14758285	BnaA03g28540D	*B. napus*	LK031800	1725145	1725208	7.4e−29	Calcium-dependent lipid-binding family protein
	Bra001103	*B. rapa*	A03	14757826	14757889	6.5e−27	
Bn_A03_p14583041	BnaA03g28150D	*B. napus*	LK031800	1898103	1898167	4.6e−29	Zinc finger CCCH domain-containing protein
Bn_A03_p14870270	BnaA03g28800D	*B. napus*	LK031800	1628327	1628431	7.3e−52	Multisubstrate pseudouridine synthase
	Bra001125	*B. rapa*	A03	14869770	14869874	6.3e−50	
Bn_A03_p14888403	BnaA03g28850D	*B. napus*	LK031800	1609368	1609522	2.6e−82	Serine/Threonine-protein kinase WNK1
Bn_A03_p14927037	BnaA03g28960D	*B. napus*	LK031800	1577297	1577397	1.1e−47	Serine/Threonine-protein kinase RBK2
	Bra001138	*B. rapa*	A03	14926686	14926786	3.6e−48	
Bn_A03_p15237693	BnaA03g29380D	*B. napus*	LK031800	1358410	1358474	1.1e−26	Zinc finger family protein
	Bra001193	*B. rapa*	A03	15237289	15237353	1.6e−29	
Bn_A03_p15265791	BnaA03g29440D	*B. napus*	LK031800	1332881	1332945	4.6e−29	HCO3-transporter family
Bn_A03_p15351982	BnaA03g29550D	*B. napus*	LK031800	1263333	1263397	1.1e−26	Alpha/beta-Hydrolases superfamily protein
Bn_A03_p15349925	BnaA03g29540D	*B. napus*	LK031800	1265409	1265562	4.2e−82	E3 ubiquitin ligase
	Bra001210	*B. rapa*	A03	15349521	15349674	3.5e−80	
Bn_A03_p15708192	Bo5g137770	*B. oleracea*	C05	42993041	42993093	6.7e−17	Peptidyl-prolyl isomerase
Bn_A03_p15704830	BnaA03g30390D	*B. napus*	LK031800	887529	887628	4.6e−50	Putative DNA repair protein
	Bra001288	*B. rapa*	A03	15704427	15704530	6.2e−53	
Bn_A03_p15906810	BnaA03g30980D	*B. napus*	LK31800	664657	664721	7.6e−26	Electron transport SCO1/SenC family protein
	Bra001335	*B. rapa*	A03	15906406	15906470	2.6e−26	
Bn_A03_p16123758	BnaA03g31520D	*B. napus*	LK031800	435047	435111	4.6e−29	Transcriptional factor B3 family protein
	Bra001387	*B. rapa*	A03	16123354	16123418	1.6e−29	
Bn_A03_p16126013	BnaA03g31530D	*B. napus*	LK031800	432849	432913	4.6e−29	Transcriptional factor B3 family protein
	Bra001387	*B. rapa*	A03	16125553	16125617	1.6e−29

**Table 6:** List of leucine rich-repeat (LRR) receptor-like kinases in the QTL genomic region associated with resistance to *Plasmodiophora brassicae* pathotypes 2F, 3H, 5I, 2B, 3D, 5G, 8E, 3O and 8P.

Gene ID	Symbol	Chromosome	Position		Description of gene functions
			Start	End	
103860116	LOC103860116	A03	14449648	14453765	LRR receptor-like serine/threonine-protein kinase RPK2
103859010	LOC103859010	A03	14678662	14682582	Probable inactive leucine-rich repeat receptor-like protein kinase At3g03770
103859018	LOC103859018	A03	14707594	14710622	Probably leucine-rich repeat receptor-like protein kinase At2g25790
103859177	LOC103859177	A03	15370285	15370993	Leucine-rich repeat extensin-like protein 1
103859225	LOC103859225	A03	15544649	15545806	Probably leucine-rich repeat receptor-like protein kinase At5g48380
103859386	LOC103859386	A03	16187253	16190261	Plant intracellular Ras-group-related LRR protein 9-like

## Discussion

In this study, the European winter *B. napus* cv. ‘Tosca’ exhibited high clubroot resistance to the *P. brassicae* pathotypes 2F, 3H, 5I, 2B and 8E, which was comparable to levels previously reported in ‘Mendel’^8,15,25^. In addition, ‘Tosca’ exhibited higher levels of resistance to pathotypes 3O and 8P, which were reported to cause increased disease (MR and S, respectively) on ‘Mendel’^8,15^. In contrast, ‘Tosca’ was susceptible to pathotypes 6M, 5X (L-G1 and L-G2), 5L, 3A, 5K and 8J, which caused only minor or moderate disease on ‘Mendel’^8,15,25^. ‘Tosca’ exhibited moderate resistance to pathotypes 8N, 3D and 5G, as opposed to the complete resistance shown by ‘Mendel’ to these pathotypes. Both ‘Tosca’ and ‘Mendel’ were susceptible to pathotype 5C. Collectively, the results of this study showed that ‘Tosca’ was resistant to 7 isolates, moderately resistant to 4, and susceptible to another 7 isolates. In contrast, ‘Mendel’ was resistant to 10 isolates, moderately resistant to 5, and susceptible to 3 isolates^8,15,25^. The differences in the resistance phenotypes of ‘Tosca’ and ‘Mendel’ to the same pathotypes in this study and in Fredua-Agyeman et al.^25^, respectively, suggest that loci controlling clubroot resistance in the two cultivars might be different. However, different loci can confer resistance to the same *P. brassicae* pathotypes.

QTL mapping is usually carried out with markers that follow expected ‘Mendelian’ segregation ratios, which in a DH population should be 1:1 for resistance and susceptibility. Xu et al. (2008)^26^ reported that QTL mapping could benefit from using all available (‘Mendelian’ + ‘distorted’) marker resources. Recently, Coulton et al.^27^ reported that markers that showed extreme segregation distortion affected the estimation of recombination between marker pairs and hence should be discarded. In this study, Chi-square goodness of test was used to measure deviation from a 1:1 ratio. By adjusting for *p*-value using the Bonferroni correction, we retained an additional 785 SNP markers for the QTL analysis. The use of the additional ‘distorted’ markers did not result in much improvement in the QTL profiles compared with the use of only the ‘Mendelian’ markers at a minimum significance threshold of *p* < 0.05 (Figs. 2 and Fig. S1). In addition, the use of the markers with lower levels of segregation distortion did not affect the order of the genetic map. Based on the ‘Mendelian’ and ‘distorted’ markers, however, the genomic region conferring resistance to the nine *P*. *brassicae* pathotypes mapped to a narrower (15.5 cM) region compared with the use of only the ‘Mendelian’ markers (20.6 cM). Therefore, as previously reported, it was beneficial for QTL mapping to include both the ‘Mendelian’ and low ‘distorted’ markers.

The genomic region identified to confer resistance to *P. brassicae* pathotypes 2F, 3H, 5I, 2B, 3D, 5G, 8E, 3O and 8P mapped to the top half of the A03 chromosome in *B. rapa* and *B. napus*. Fredua-Agyeman et al.^18,28^ positioned the *CRk*^29^, *Crr3*^30,31^ and the *CRd*^32^ genes to the top half of the A03 chromosome in *B. rapa* and *B. napus*. The physical position of these genes spans a genomic region of approximately 765 Kb (14,396,950–15,161,430 nt) on the *B. rapa* genome^28^ and 1731 Kb (24,338,876–26,070,712 nt) on the *B. napus* genome^18^. The QTL region identified in this study spanned 837.6 to 946.6 Kb, consistent with the values obtained in our previous studies.

A closer inspection of the QTL region indicated that the different-sized fragments of the A03 chromosome were responsible for the resistance to the different *P. brassicae* pathotypes (Fig. 3). By use of the ‘Mendelian’ and ‘distorted’ markers, the genomic region of the QTL could be partitioned into at least two CR ‘hotspots’. The first CR hotspot comprised the region between the SNP markers Bn_A03_p14758285 (57.9 cM) and Bn_A03_p15004059 (64.8 cM), while the second comprised the region between the SNP markers Bn_A03_p14968153 (67.0 cM) to Bn_A03_p15351982 (73.4 cM). The first hotspot conferred resistance to all nine (2F, 3H, 5I, 2B, 3D, 5G, 8E, 3O and 8P) aforementioned pathotypes while the second conferred resistance to pathotypes 2B, 3D, 5G, 8E and 8P. In the case of the use of only the ‘Mendelian’ markers, three CR hotspots could be delimited. The first hotspot, between the SNP markers Bn_A03_p14784764 (56.6 cM) to Bn_A03_p14927037 (63.7 cM), conferred resistance to pathotypes 2F, 2B, 3D, 5G and 3O. The second CR hotspot, between SNP markers Bn_A03_p15149454 (63.7 cM) to Bn_A03_p14968153 (67.0 cM), conferred resistance to all nine pathotypes. The third CR hotspot, between SNP markers Bn_A03_p15237693 (69.7 cM) and Bn_A03_p15704830 (77.2 cM), conferred resistance to 8 of the 9 pathotypes, with the exception of pathotype 2B.

Disease resistance is a complex trait and may involve the interaction of subunits of the same gene or different genes. In this study, the QTL region contained several genes including LRR kinases. Mutation studies in Arabidopsis showed that the interaction between the LRR and the kinase domains of the ERECTA (ER) gene were required for resistance to rot caused by *Plectosphaerella cucumerin*^33^. In addition, the QTL region identified in this study contained transcriptional factor family proteins. In rice, the interaction of transcription activator-like effector (TALE) proteins and transcription factor IIA small subunit was reported to determine resistance or susceptibility to bacterial leaf blight and bacterial leaf streak^34^. Epistatic interaction between the CR genes *CRa*/*CRb*^Kato^ on the A03 chromosome and the *Crr1* genes on the A08 chromosome of *B. rapa* and *B. napus* was reported to confer clubroot resistance in *Brassica*^35^. The fact that the QTL region contained several genes suggests the possibility of various interactions within subunits of the same genes and also amongst different genes. This is further complicated by the identification of the CR genes *CRk*^29^, *Crr3*^30,31^ and *CRd*^32^ in the QTL region introgressed from ‘Tosca’. Therefore, mutation studies are needed to confirm whether the CR gene(s) introgressed from ‘Tosca’ are three different genes or alleles of the same gene.

The genome region conferring clubroot resistance derived from ‘Mendel’ was reported by Fredua-Agyeman and Rahman^18^ to be located on the A03 chromosome at positions 24,376, 817 to 24,684,311b in *B. rapa* and 40,936,414 to 41,929,968 b in *B. napus*. The CR loci from ‘Mendel’ conferred resistance against the old *P. brassicae* pathotypes 2F, 3H, 5I, 6M and 8N from Alberta, Canada. In contrast, the CR loci derived from ‘Tosca’ in this study was located upstream (14,396,950–15,161,430 nt in *B. rapa* and 24,338,876–26,070,712 nt in *B. napus*) of the genomic region confering clubroot resistance in ‘Mendel’. Thus, the genomic hotspot regions reported in ‘Tosca’ (this study) and that reported in ‘Mendel’^18^ are different.

In conclusion, the Swedish *B. napus* cv. ‘Tosca’ is resistant to multiple *P. brassicae* pathotypes, including isolates representing the ‘old’ pathotypes 2F, 3H and 5I as well as the ‘new’ pathotypes 2B, 3O, 8E and 8P. This host also exhibited moderate resistance to isolates representing the ‘old’ pathotype 8N and the ‘new’ pathotypes 3D and 5G. Unfortunately, ‘Tosca’ was susceptible to isolates representing the ‘old’ pathotype 6M and the ‘new’ pathotypes 5X (L-G1 and L-G2), 5L, 3A, 5K and 8J. This is the first report on the genomic loci controlling clubroot resistance in the *Brassica napus* cv. ‘Tosca”. The resistance was shown to be different from the clubroot resistance derived from ‘Mendel’. The increased clubroot severity on ‘Tosca’, especially in response to pathotypes 3A and 3D, which constitute the bulk of the virulent pathotypes (note: pathotype 3H is still most prevalent of all pathotypes) in Alberta, makes the cultivar unattractive as the sole CR donor in the breeding of commercial canola varieties in Canada. However, the *CRk*, *Crr3* and *CRd* gene(s) present in ‘Tosca’ could be stacked with other CR genes present in additional resistance resources such as ‘Mendel’, ECD 02 and ECD 04.

## Materials and methods

### Plant materials

One hundred sixteen doubled haploid (DH) lines obtained from F_1_ plants of the cross ‘11SR0099’ (clubroot resistant) × ‘12DH0001’ (clubroot susceptible) were used as the mapping population. The CR parent ‘11SR0099’ is a spring-type canola line derived from a spring canola × winter canola cv. ‘Tosca’ cross, while the CS parent ‘12DH0001’ is a spring-type canola line with good agronomy and quality characteristics. Seeds of the DH parents and lines were provided by Corteva AgriScience (Caledon, ON, Canada), while seeds of a ‘Tosca’, used as a resistant (negative) control in the inoculation experiments, were obtained from Prof. Ann-Charlotte Wallenhammar (Swedish University of Agricultural Sciences, Skara, Sweden). The European Clubroot Differential (ECD) 04 (*B. rapa* subsp. *rapifera*), which exhibits broad-spectrum resistance to many Canadian isolates of *P. brassicae*^25^, was included as resistant (negative) control in the inoculation experiments, while ECD 05 (*B. rapa* var. *pekinensis* ‘Granaat’)^36^ and *B. napus* cv. ‘Westar’^25^ were included as susceptible (positive) controls. The collection of plant materials and all conducted experiments complied with relevant guidelines/regulations of the University of Alberta, Canada and International Treaty for Plant Genetic Resources guidelines and legislation.

### Pathogen isolates and inoculum preparation

Eighteen *P. brassicae* isolates were used in inoculation experiments under controlled conditions in the greenhouse. These consisted of five single-spore isolates (SSIs) (SACAN-ss3, SACAN-ss1, ORCA-ss4, AbtJE-ss1 and ORCA-ss2), classified as pathotypes 2F, 3H, 5I, 6M and 8N, respectively, on the Canadian Clubroot Differential (CCD) set^15^, and 13 field isolates (L-G1 + L-G2, D-G3, F183-14, F3-14, F1-14, CDCN#6, F187-14, F175-14, F12DH00015, F10-15, F381-16/C.C. and UofA/County#37), classified as pathotypes 5X, 5L, 2B, 3A, 3D, 5G, 8E, 5C, 8J, 5K, 3O and 8P, respectively^15^. Two of the field isolates, L-G1 and L-G2^8^, represented the same pathotype (5X). The 18 isolates (representing isolates of 17 pathotypes) were maintained as frozen (− 20 °C) root galls of the universally susceptible host ECD 05 until needed. Clubroot inoculum was prepared following Fredua-Agyeman et al.^25^ by macerating root galls using a variable-speed blender, filtering the spore suspension through three layers of cheesecloth, and adjusting the final resting spore concentration to 1 × 10^7^ spores/mL with sterile distilled water. Each batch of inoculum was stored at 4 °C and used within 24 h after preparation.

### Evaluation of DH lines for clubroot resistance

Inoculation experiments were performed in two greenhouses at the at the Crop Diversification Centre North (CDCN), Alberta Agriculture and Forestry (AAF), Edmonton, Canada. Briefly, 15–20 seeds of the DH parents (‘11SR0099’ and ‘12DH0001’), each DH line, ‘Tosca’, ECD 04, ECD 05 and ‘Westar’, were placed on moistened Whatman No. 1 filter paper in Petri dishes and kept at room temperature under natural light^20^. After 7 days, 8–12 seedlings of each genotype were inoculated by dipping their entire roots in a resting spore suspension for about 10–20 s^37^ and then transplanted into 13 × 13 × 15 cm (L × B × D) pots filled with Sunshine Mix #4 Aggregate Plus soilless Mix (Sungro Horticulture Canada Ltd)^25,38^. To minimize disease escape, 1 mL of additional inoculum was dispensed into the potting mixture surrounding each seedling with a micropipette^39^. The plants were kept in the greenhouse maintained under a 16 h photoperiod and day and night temperatures of 20–25 °C and 15–18 °C, respectively. The pots were arranged in a randomized complete block design (RCBD) in 225 × 104 × 20 cm (L × B × D) trays^38^. The potting mixture were kept saturated with slightly acidic water (pH ≈ 5.5–6.9) for the first 2 weeks, and then watered daily and fertilized every 2 weeks with 200 ppm Plant Prod 20–20–20 (N–P–K) with micronutrients (Plant Products, Leamington, ON, Canada).

Eight weeks after inoculation, the plants were uprooted, washed and assessed for disease severity on a 0–3 scale^40^, where: 0 = no galling, 1 = a few small galls on the lateral roots, 2 = moderate galling on the lateral roots but not on the main root, and 3 = severe galling on both the lateral and main root. Disease severity symptoms, measured as an index of disease (ID, 0–100%), were calculated following Strelkov et al.^20^ as shown below:

$${\text{ID}}~\left( \% \right) = \frac{{\sum \left( {n \times 0 + ~n \times 1 + n \times 2 + n \times 3} \right)}}{{N \times 3}} \times 100$$

where *n* = number of plants in each symptom severity class and $N$ = is the total number of plants. The inoculation experiments were repeated two times. Based on the mean ID values of the combined data ± the standard error (SEM), the DH lines were classified as resistant (R) (Mean ID ± SEM ≤ 30%), moderately resistant (MR) (30% < Mean ID ± SEM ≤ 50%) or susceptible (S) (Mean ID ± SEM > 50%) as per the recommendations of the Western Canada Canola/Rapeseed Recommending Committee (WCC/RCC)^38^.

### Statistical analyses of phenotypic data

Statistical analyses of the disease severity data for the individual experiments and the combined data were conducted with SAS v. 9.4 (SAS Institute, United States) as described by Fredua-Agyeman et al.^25,38^. In brief, the PROC CORR function was used to determine the correlation among the mean ID values for each DH line, parents and checks for each pathotype in the three experiments. Broad sense heritability (*H*^2^), which is the ratio of total genetic variance to phenotypic variance, was estimated from variance components from Analysis of Variance (ANOVA)^41,42^. The PROC MEANS function was used to calculate the mean ID, standard error of the mean (SEM), minimum and maximum ID for each genotype and isolate investigated. The PROC FREQ function was used to count the number of accessions that were resistant (Mean ID ± SEM ≤ 30%), moderately resistant (30 < ID ± SEM ≤ 50%) or susceptible (ID ± SEM > 50%) to each *P. brassicae* isolate based on the combined data, while SigmaPlot (SYSTAT Software Inc., San Jose, California, USA) was used to create histograms. The Shapiro–Wilk test was used to test for normality in the phenotypic data^43^. The 1R: 1S ratio suggested for segregation in a DH population was determined through Chi-Square goodness of fit tests (χ^2^) at *p* ≤ 0.05 for each of the 18 *P*. *brassicae* isolates. Differences in the mean ID values of all DH lines to pairs of the 18 *P. brassicae* isolates were compared with Tukey’s test at *p* ≤ 0.05.

### Genotyping with SNP markers

The parental lines and the DH population were genotyped using the *Brassica* 15 K array, which contained 13,714 SNPs, at SGS TraitGenetics GmbH, Gatersleben, Germany, according to the manufacturer’s protocols^44^. The software TASSEL v5.2.2.5^45^ was used to perform SNP filtering by deleting failed SNP reactions, setting minor allele frequency (MAF) to ≤ 0.05 and removing markers missing data for > 5% of the accessions. Segregation distortion was determined through a χ^2^ test for goodness-of-fit for the 1:1 ratio expected for a DH population. A minimum significance threshold of *p* < 0.05 was used for markers that followed expected ‘Mendelian’ ratio, while adjusted Bonferroni correction *p-*values (α/*n*, where α = level of significance, *n* = number of markers) were used to select markers with ‘minor’ segregation distortion^46^. Markers that showed extreme segregation distortion were discarded.

### Construction of genetic linkage maps

Two ‘draft’ linkage maps were constructed using the minimum spanning tree map (MSTMap) program with the following parameters: logarithm of odds (LOD) value of 10.0, a maximum distance between markers of 15 cM and Kosambi mapping function^47,48^. The first linkage map was constructed only with markers that fit the expected 1: 1 Mendelian ratio, while the second linkage map was constructed using markers that fit the 1:1 Mendelian ratio expected for a DH population and markers that showed ‘minor’ segregation distortion. In both cases, markers that mapped to the same position were placed in the same bin, with only one of the markers retained for linkage analysis. MAPMAKER/EXP v. 3.0b^49^ was run at a logarithm of odds (LOD) score ≥ 3.0 and recombination fraction (ϴ) value ≤ 0.40 to ‘refine’ the marker order obtained by the MSTMap software. Recombination fractions were converted to centiMorgans (cM) using the Kosambi mapping function^47^. Linkage groups were assigned to chromosomes based on the SNP sequence information provided by SGS TraitGenetics GmbH.

### Additive effect QTL mapping

Quantitative trait loci (QTL) mapping was conducted separately with only the ‘Mendelian’ markers and with ‘Mendelian’ and ‘distorted’ markers together. The QTL analyses were carried out by composite interval mapping (CIM)^50^ using WinQTL Cartographer v. 2.5^51^. The program was run at a walking speed of 1 cM and with the following settings: forward–backward regression method, a window size 2.0 cM, five background markers as cofactors, 1000 permutations and *p* < 0.05. The significance level required to declare a QTL was set at LOD ≥ 5.0. Locations of putative QTL were estimated based on two-LOD support intervals for a 99% confidence interval (CI)^52^.

The QTL designations were of the order genus (1 letter), species (1 letter), genome (1 letter), chromosome number (1 letter), pathotype name (3 letters), closest published gene(s) (3–6 letters) and QTL number (2 letters)^35,53^. The percentage of phenotypic variation (*R*^2^) explained by each QTL was calculated. QTL were arbitrary assigned as major-, moderate- or minor-effect QTL when the *R*^2^ explained > 50%, 25–50% or < 25% of the phenotypic variation, respectively. The additive effects of each QTL were calculated by deducting the phenotypic average of all individuals with the susceptible DH parent allele from all individuals with the resistant DH parent allele.

### Identification of candidate genes

The physical positions of the SNP markers in the QTL CI region were mapped to the *B*. *napus*, *B. rapa* and *B*. *oleracea* reference genomes deposited in the National Center for Biotechnology Information (NCBI) GenBank database (www.ncbi.nlm.nih.gov). Candidate genes present in the QTL two-LOD confidence interval were identified by BlastN searches (E-value ≤ E−20, minimum identity of sequence ≥ 95%) of the three *Brassica* genome sequences.

### Ethical approval

On behalf of all authors, the corresponding author states that the collected seeds and all conducted experiments in this study complied with relevant guidelines/regulations of the University of Alberta, the Canada Food Inspection Agency and International Treaty for Plant Genetic Resources guidelines and legislation.

## Supplementary Information

Supplementary Information.

## Data Availability

The datasets generated during the current study are available in the manuscript or the supplementary materials. The SNP used for genotyping is available can be downloaded from https://static-content.springer.com/esm/art%3A10.1007%2Fs00122-016-2746-7/MediaObjects/122_2016_2746_MOESM3_ESM.pdf or from Clark et al.^44^
