# Supplementary material for: Clubroot resistance derived from the European Brassica napus cv. ‘Tosca’ is not effective against virulent Plasmodiophora brassicae isolates from Alberta, Canada
Source: Sci Rep. 2021 Jul 14;11:14472. doi: 10.1038/s41598-021-93327-0 (PMC8280172; doi:10.1038/s41598-021-93327-0)
Supplement: Supplementary file 1 — Supplementary Information. [file 41598_2021_93327_MOESM1_ESM.docx]

**Table S1.** Heritability (*H*) of clubroot resistance based on the mean index of disease of each doubled haploid line obtained from F_1_ plants of the cross between ‘11SR0099’ (clubroot resistant) × ‘12DH0001’ (clubroot susceptible) for individual greenhouse experiments and the combined data and correlation between individual experiments

| Pathotype | Heritability greenhouse experiments | | | | Pearson correlation co-efficient (*r*) (range, sig.) |
| --- | --- | --- | --- | --- | --- |
|  | 1 | 2 | 3 | Combined |  |
| 2F | 0.9731 | 0.9633 | 0.9597 | 0.9579 | 0.9341-0.9410, *P*<0.0001 |
| 3H | 0.9926 | 0.9917 | 0.9943 | 0.9926 | 0.9444-0.9485, *P*<0.0001 |
| 5I | 0.9406 | 0.9522 | 0.9650 | 0.9394 | 0.7989-0.8823, *P*<0.0001 |
| 6M | 0.6578 | 0.6798 | 0.6705 | 0.5768 | 0.5955-0.8110, *P*<0.0001 |
| 8N | 0.8849 | 0.8937 | 0.9027 | 0.8861 | 0.9168-0.9195, *P*<0.0001 |
| 5X (L-G1) | 0.7643 | 0.7840 | 0.8071 | 0.7568 | 0.7392-0.8531, *P*<0.0001 |
| 5X (L-G2) | 0.8770 | 0.7622 | 0.8019 | 0.7799 | 0.6648-0.7291, *P*<0.0001 |
| 5L (D-G3) | 0.7284 | 0.6769 | 0.6369 | 0.6198 | 0.6122-0.6719, *P*<0.0001 |
| 2B | 0.9707 | 0.9891 | 0.9713 | 0.9758 | 0.9518-0.9739, *P*<0.0001 |
| 3A | 0.8933 | 0.9987 | 0.9237 | 0.8980 | 0.9281-0.9572, *P*<0.0001 |
| 3D | 0.9993 | 0.9717 | 0.9993 | 0.9725 | 0.9541-0.9737, *P*<0.0001 |
| 5C | 0.9304 | 0.9264 | 0.9989 | 0.9320 | 0.9379-0.9636, *P*<0.0001 |
| 5G | 0.9520 | 0.9391 | 0.9345 | 0.9338 | 0.8783-0.9237, *P*<0.0001 |
| 8E | 0.9821 | 0.9790 | 0.9762 | 0.9809 | 0.9715-0.9847, *P*<0.0001 |
| 5K | 0.7859 | 0.7696 | 0.7207 | 0.7309 | 0.6339-0.8087, *P*<0.0001 |
| 8J | 0.8073 | 0.7308 | 0.7737 | 0.7482 | 0.8211-0.9033, *P*<0.0001 |
| 3O | 0.9860 | 0.9834 | 0.9868 | 0.9994 | 0.9639-0.9715, *P*<0.0001 |
| 8P | 0.9898 | 0.9837 | 0.9876 | 0.9901 | 0.9694-0.9818, *P*<0.0001 |

*Plasmodiophora brassicae* pathotypes 2F, 3H, 5I, 6M and 8N are single-spore isolates (Strelkov et al. 2005; Xue et al 2008). Pathotypes 5X (L-G1 and L-G2) and 5L are field isolates collected in 2013 (Strelkov et al. 2016a, 2018). Pathotypes 2B, 3A, 3D, 5C, 5G and 8E are field isolates collected in 2014 (Strelkov et al. 2015, 2018). Pathotypes 5K and 8J are field isolates collected in 2015 (Strelkov et al. 2016b, 2018). Pathotypes 3O and 8P are field isolates collected in 2016 (Strelkov et al. 2017, 2018).

**Table S2.** Pairwise comparison of 18 *Plasmodiophora brassicae* pathotypes based on differences in mean index of disease (ID) values of doubled haploid lines obtained from F_1_ plants of the cross ‘11SR0099’ (clubroot resistance derived from the *B. napus* cv. ‘Tosca’) × ‘12DH0001’ (clubroot susceptible)

| Pathotypes | 2F | 3H | 5I | 6M | 8N | 5X (L-G1) | 5X (L-G2) | 5L | 2B | 3A | 3D | 5C | 5G | 8E | 5K | 8J | 3O | 8P |
| --- | --- | --- | --- | --- | --- | --- | --- | --- | --- | --- | --- | --- | --- | --- | --- | --- | --- | --- |
| 2F |  |  |  |  |  |  |  |  |  |  |  |  |  |  |  |  |  |  |
| 3H |  |  |  |  |  |  |  |  |  |  |  |  |  |  |  |  |  |  |
| 5I |  | *** |  |  |  |  |  |  |  |  |  |  |  |  |  |  |  |  |
| 6M | *** | *** | *** |  |  |  |  |  |  |  |  |  |  |  |  |  |  |  |
| 8N | *** | *** |  |  |  |  |  |  |  |  |  |  |  |  |  |  |  |  |
| 5X (L-G1) | *** | *** | *** |  |  |  |  |  |  |  |  |  |  |  |  |  |  |  |
| 5X (L-G2) | *** | *** |  |  |  |  |  |  |  |  |  |  |  |  |  |  |  |  |
| 5L | *** | *** | *** |  |  |  |  |  |  |  |  |  |  |  |  |  |  |  |
| 2B |  |  |  | *** | *** | *** | *** | *** |  |  |  |  |  |  |  |  |  |  |
| 3A | *** | *** | *** |  |  |  |  |  | *** |  |  |  |  |  |  |  |  |  |
| 3D |  |  |  | *** | *** | *** | *** | *** |  | *** |  |  |  |  |  |  |  |  |
| 5C | *** | *** |  |  |  |  |  |  | *** |  | *** |  |  |  |  |  |  |  |
| 5G |  | *** |  |  |  | *** |  | *** |  |  |  |  |  |  |  |  |  |  |
| 8E |  |  |  | *** | *** | *** | *** | *** |  | *** |  | *** |  |  |  |  |  |  |
| 5K | *** | *** | *** |  |  |  |  |  | *** |  | *** |  | *** | *** |  |  |  |  |
| 8J | *** | *** | *** |  |  |  |  |  | *** |  | *** |  | *** | *** |  |  |  |  |
| 3O |  |  |  | *** | *** | *** | *** | *** |  | *** |  | *** |  |  | *** | *** |  |  |
| 8P |  |  |  | *** | *** | *** | *** | *** |  | *** |  | *** | *** |  | *** | *** |  |  |

*Plasmodiophora brassicae* pathotypes 2F, 3H, 5I, 6M and 8N are single-spore isolates (Strelkov et al. 2005; Xue et al 2008). Pathotypes 5X (L-G1 and L-G2) and 5L are field isolates collected in 2013 (Strelkov et al. 2016a, 2018). Pathotypes 2B, 3A, 3D, 5C, 5G and 8E are field isolates collected in 2014 (Strelkov et al. 2015, 2018). Pathotypes 5K and 8J are field isolates collected in 2015 (Strelkov et al. 2016b, 2018). Pathotypes 3O and 8P are field isolates collected in 2016 (Strelkov et al. 2017, 2018).

*** Comparisons between pathotypes significant at *P* < 0.05

**Table S3**. The distribution of 2253 ‘Mendelian’ single-nucleotide polymorphism (SNP) markers on 24 linkage groups representing 18 of the 19 chromosomes used to map the QTL for clubroot resistance in doubled haploid lines derived from the *Brassica napus* cv ‘Tosca’

| Chromosome | Linkage group | Number of SNP markers used for QTL mapping | | Total map length/ (cM) | Marker density/loci |
| --- | --- | --- | --- | --- | --- |
|  |  | Actual | Bin^∆^ |  |  |
| A01 | 1 | 187 | 68 | 145.9 | 1.3 |
| A02 | 2 | 61 | 23 | 74.3 | 0.8 |
| A03a | 3 | 135 | 65 | 163.4 | 0.8 |
| A03b | 4 | 35 | 13 | 13.1 | 2.7 |
| A03c | 5 | 12 | 7 | 17.9 | 0.7 |
| A04 | 6 | 129 | 65 | 170.8 | 0.8 |
| A05 | 7 | 5 | 3 | 68.4 | 0.1 |
| A06 | 8 | 204 | 84 | 181.9 | 1.1 |
| A07 | 9 | 158 | 49 | 76.7 | 2.1 |
| A08 | 10 | 192 | 49 | 128.7 | 1.5 |
| A09a | 11 | 45 | 19 | 83.4 | 0.5 |
| A09b | 12 | 57 | 35 | 68.7 | 0.8 |
| A10 | 13 | 132 | 44 | 89.2 | 1.5 |
| C01a | 14 | 117 | 23 | 64.4 | 1.8 |
| C01b | 15 | 18 | 5 | 19.8 | 0.9 |
| C02 | 16 | 104 | 32 | 119.4 | 0.9 |
| C03 | 17 | 163 | 45 | 149.1 | 1.1 |
| C04 | 18 | 22 | 8 | 18.5 | 1.2 |
| C05 | 19 | 103 | 58 | 152.4 | 0.7 |
| C07 | 20 | 178 | 58 | 189.4 | 0.9 |
| C08a | 21 | 97 | 34 | 72.3 | 1.3 |
| C08b | 22 | 26 | 12 | 25.6 | 1.0 |
| C08c | 23 | 19 | 6 | 60.4 | 0.3 |
| C09 | 24 | 54 | 24 | 57.8 | 0.9 |
| Total or Average | | 2253 | 829 | 2211.5 | 1.0 |

^∆^ Multiple markers that mapped to the same position on the linkage map were put in the same bin

**Table S4**. Summary of QTL on chromosome A03 associated with clubroot resistance in doubled haploid lines derived from the *Brassica napus* cv. ‘Tosca’ inoculated with different *Plasmodiophora brassicae* pathotypes using ‘Mendelian’ markers

| Identified QTL | Pathotype | Expt | QTL pisitions (cM) ^∆^ | | Left SNP Marker | Right SNP marker | LOD | Additive | R^2^ (%) |
| --- | --- | --- | --- | --- | --- | --- | --- | --- | --- |
|  |  |  | Peak | Conf Interval |  |  |  |  |  |
| *Bn.A3P2F.Crr3/CRk/CRd 1.1* | 2F | 1 | 30.7 | 29.5-32.8 | Bn_A03_p14968153 | Bn_A03_p15708192 | 14.3 | -34.0 | 20.8 |
|  |  | 2 | 27.8 | 25.9-32.8 | Bn_A03_p14784764 | Bn_A03_p15708192 | 17.8 | -24.1 | 24.1 |
|  |  | 3 | 30.7 | 29.5-32.8 | Bn_A03_p14968153 | Bn_A03_p15708192 | 19.9 | -31.8 | 34.4 |
|  |  | Pooled | 30.7 | 29.5-32.8 | Bn_A03_p14968153 | Bn_A03_p15708192 | 22.1 | -26.8 | 26.8 |
| *Bn.A3P3H.Crr3/CRk/CRd 1.1* | 3H | 1 | 31.7 | 31.0-32.7 | Bn_A03_p15149454 | Bn_A03_p15708192 | 29.6 | -33.9 | 50.8 |
|  |  | 2 | 31.7 | 30.7-34.4 | Bn_A03_p15149454 | Bn_A03_p15704830 | 37.5 | -38.7 | 93.3 |
|  |  | 3 | 31.7 | 30.7-33.4 | Bn_A03_p15149454 | Bn_A03_p15708192 | 37.4 | -39.4 | 90.6 |
|  |  | Pooled | 31.7 | 31.3-33.3 | Bn_A03_p15149454 | Bn_A03_p15708192 | 42.1 | -37.2 | 92.5 |
| *Bn.A3P5I.Crr3/CRk/CRd 1.1* | 5I | 2 | 30.7 | 30.4-32.9 | Bn_A03_p15149454 | Bn_A03_p15708192 | 11.0 | -28.6 | 28.4 |
|  |  | 3 | 30.7 | 28.6-33.0 | Bn_A03_p14968153 | Bn_A03_p15708192 | 16.1 | -26.8 | 35.5 |
|  |  | Pooled | 30.7 | 29.1-32.9 | Bn_A03_p14968153 | Bn_A03_p15708192 | 10.1 | -26.4 | 17.7 |
| *Bn.A3P2B.Crr3/CRk/CRd 1.1* | 2B | 1 | 16.7 | 15.7-17.7 | Bn_A03_p14355646 | Bn_A03_p14583041 | 19.3 | -36.1 | 56.0 |
|  |  | Pooled | 16.7 | 15.7-18.3 | Bn_A03_p14355646 | Bn_A03_p14583041 | 24.1 | -41.5 | 73.6 |
| *Bn.A3P2B.Crr3/CRk/CRd 1.2* | 2B | 2 | 27.1 | 25.8-27.4 | Bn_A03_p14784764 | Bn_A03_p14968153 | 25.2 | -43.5 | 72.9 |
|  |  | 3 | 27.1 | 25.9-27.3 | Bn_A03_p14784764 | Bn_A03_p14968153 | 26.5 | -41.5 | 76.5 |
| *Bn.A3P3D.Crr3/CRk/CRd 1.1* | 3D | 1 | 20.0 | 19.4-21.4 | Bn_A03_p14611641 | Bn_A03_p14888403 | 11.2 | -32.9 | 15.9 |
|  |  | Pooled | 20.0 | 19.4-23.1 | Bn_A03_p14611641 | Bn_A03_p14758285 | 10.4 | -30.8 | 14.6 |
| *Bn.A3P3D.Crr3/CRk/CRd 1.2* | 3D | 1 | 31.7 | 29.6-32.9 | Bn_A03_p14968153 | Bn_A03_p15708192 | 13.8 | -33.6 | 24.3 |
|  |  | 2 | 31.7 | 30.7-32.7 | Bn_A03_p15149454 | Bn_A03_p15708192 | 10.4 | -37.8 | 29.2 |
|  |  | 3 | 30.7 | 29.4-32.7 | Bn_A03_p14968153 | Bn_A03_p15708192 | 23.7 | -30.4 | 69.3 |
|  |  | Pooled | 30.7 | 28.9-32.9 | Bn_A03_p14968153 | Bn_A03_p15708192 | 12.4 | -32.1 | 16.6 |
| *Bn.A3P3D.Crr3/CRk/CRd 1.3* | 3D | 2 | 37.0 | 35.5-38.8 | Bn_A03_p15708192 | Bn_A03_p15237693 | 12.0 | -33.0 | 27.8 |
|  |  | Pooled | 36.0 | 35.0-38.0 | Bn_A03_p15708192 | Bn_A03_p15237693 | 6.8 | -27.6 | 12.9 |
| *Bn.A3P5G.Crr3/CRk/CRd 1.1* | 5G | 1 | 30.7 | 29.3-33.5 | Bn_A03_p14968153 | Bn_A03_p15708192 | 8.3 | -20.6 | 34.1 |
|  |  | 2 | 30.7 | 26.3-37.8 | Bn_A03_p14784764 | Bn_A03_p15237693 | 9.4 | -20.6 | 37.8 |
|  |  | 3 | 30.7 | 23.6-38.8 | Bn_A03_p14885241 | Bn_A03_p15237693 | 7.5 | -18.9 | 31.3 |
|  |  | Pooled | 30.7 | 24.6-38.0 | Bn_A03_p14758285 | Bn_A03_p15237693 | 9.1 | -20.1 | 36.6 |
| *Bn.A3P8E.Crr3/CRk/CRd 1.1* | 8E | 1 | 31.7 | 30.7-33.1 | Bn_A03_p15149454 | Bn_A03_p15708192 | 29.0 | -41.7 | 45.2 |
|  |  | 2 | 31.7 | 30.7-32.7 | Bn_A03_p15149454 | Bn_A03_p15708192 | 35.9 | -41.7 | 45.8 |
|  |  | 3 | 31.7 | 30.7-33.0 | Bn_A03_p15149454 | Bn_A03_p15708192 | 30.1 | -40.9 | 46.7 |
|  |  | Pooled | 31.7 | 30.7-33.2 | Bn_A03_p15149454 | Bn_A03_p15708192 | 29.8 | -40.9 | 44.4 |
| *Bn.A3P8E.Crr3/CRk/CRd 1.2* | 8E | 1 | 37.0 | 35.9-38.6 | Bn_A03_p15704830 | Bn_A03_p15237693 | 31.9 | -42.0 | 45.6 |
|  |  | 2 | 37.0 | 35.9-38.0 | Bn_A03_p15704830 | Bn_A03_p15237693 | 38.9 | -42.0 | 46.2 |
|  |  | 3 | 37.0 | 36.0-38.3 | Bn_A03_p15704830 | Bn_A03_p15237693 | 34.9 | -42.5 | 46.7 |
|  |  | Pooled | 37.0 | 35.9-38.0 | Bn_A03_p15704830 | Bn_A03_p15237693 | 33.5 | -41.4 | 45.3 |
| *Bn.A3P3O.Crr3/CRk/CRd 1.1* | 3O | 3 | 27.8 | 25.8-29.4 | Bn_A03_p14758285 | Bn_A03_p14927037 | 18.9 | -41.8 | 30.4 |
| *Bn.A3P3O.Crr3/CRk/CRd 1.2* | 3O | 1 | 31.7 | 30.7-33.4 | Bn_A03_p15149454 | Bn_A03_p15708192 | 21.3 | -42.1 | 43.6 |
|  |  | 2 | 31.7 | 30.7-33.4 | Bn_A03_p15149454 | Bn_A03_p15708192 | 16.9 | -39.6 | 34.8 |
|  |  | Pooled | 30.7 | 29.5-32.7 | Bn_A03_p14968153 | Bn_A03_p15708192 | 20.0 | -41.5 | 33.5 |
| *Bn.A3P3O.Crr3/CRk/CRd 1.3* | 3O | 1 | 37.0 | 35.7-38.4 | Bn_A03_p15708192 | Bn_A03_p15237693 | 21.0 | -42.1 | 43.6 |
|  |  | 2 | 37.0 | 35.8-38.0 | Bn_A03_p15704830 | Bn_A03_p15237693 | 19.2 | -38.3 | 36.1 |
|  |  | 3 | 36.0 | 35.0-38.1 | Bn_A03_p15708192 | Bn_A03_p15237693 | 15.8 | -40.3 | 34.9 |
|  |  | Pooled | 37.0 | 35.5-38.1 | Bn_A03_p15708192 | Bn_A03_p15237693 | 19.3 | -40.7 | 41.9 |
| *Bn.A3P8P.Crr3/CRk/CRd 1.1* | 8P | 1 | 31.7 | 31.6-33.2 | Bn_A03_p15004059 | Bn_A03_p15708192 | 48.1 | -43.2 | 95.0 |
|  |  | 2 | 31.7 | 30.7-32.7 | Bn_A03_p15149454 | Bn_A03_p15708192 | 22.0 | -41.1 | 26.4 |
|  |  | 3 | 31.7 | 31.1-32.7 | Bn_A03_p15149454 | Bn_A03_p15708192 | 40.0 | -40.8 | 90.2 |
|  |  | Pooled | 31.7 | 31.3-33.4 | Bn_A03_p15149454 | Bn_A03_p15708192 | 43.1 | -41.5 | 92.7 |

^∆^ QTL positions based on two-LOD support intervals for 99% confidence interval (CI), (Lander and Botstein, 1989)


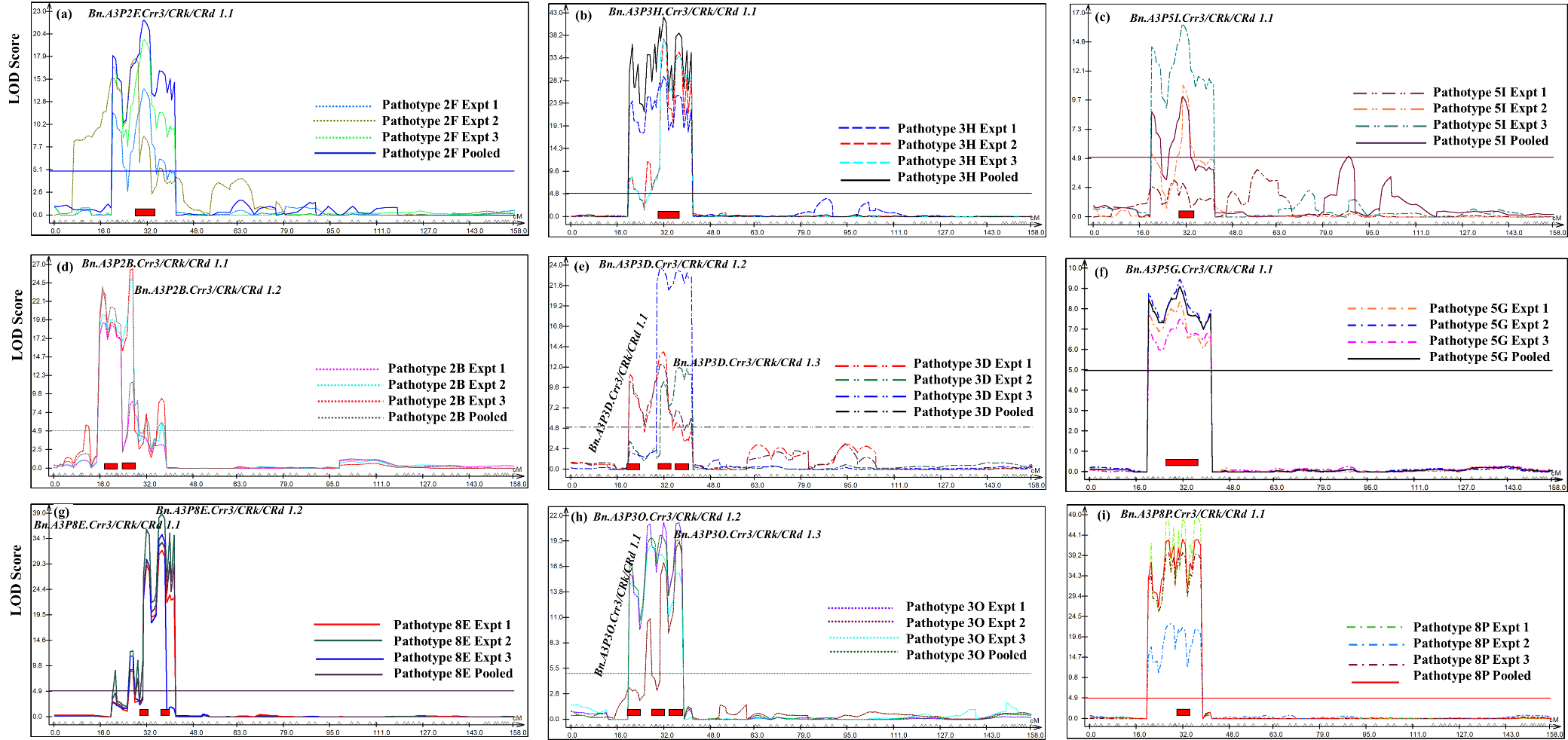


**Fig. S1.** QTL likelihood profile of the A03 chromosome of *Brassica napus* obtained by use of only ‘Mendelian’ markers. The peak regions indicate genomic regions conferring resistance to nine *Plasmodiophora brassicae* pathotypes, 2F (a), 3H (b), 5I (c), 2B (d), 3D (e), 5G (f), 8E (g), 3O (h) and 8P (i). Clubroot resistance in the DH lines was derived from the *Brassica napus* cv. ‘Tosca’. The LOD scores are indicated on the y-axis and the QTL names positioned at the peak of each profile.
